# The medical autopsy as quality assurance tool in clinical medicine: dreams and realities

**DOI:** 10.1007/s00428-015-1833-5

**Published:** 2015-08-28

**Authors:** Jan G. van den Tweel, Christian Wittekind

**Affiliations:** Department of Pathology, University Medical Center Utrecht, H4-312, 3584 CX, Utrecht, The Netherlands; Institut für Pathologie, Universitätsklinikum, Leipzig, Germany

**Keywords:** Autopsy, Quality tool, History, Future

## Abstract

The purpose of medical autopsy has changed to issues of quality assurance today. In addition, autopsies are considered valuable in medical education, e.g., delivering cases for problem-based learning for students. Many studies underscore the need for autopsies also in the era of technical progress emphasizing the continuing discrepancies between antemortem and post mortem diagnoses. Despite these important tasks, we face a decline of autopsy for several reasons with complex interactions. The role of all persons involved in this decline is evaluated and suggestions for changes are proposed. Last but not least, the future of the autopsy is in the hands of pathology itself.

## Introduction

“Dreams and realties” is the subtitle of this paper on the future of the autopsy. With the strong decline of the autopsy in the last decennia, it is a (last?) attempt to re-emphasize the importance of the autopsy in current medical practice. The dream of some pathologists is a revival of the situation of the 1970–1980s, when an autopsy was as a rule the final procedure before a patient’s file was closed. Others are content with the present situation since autopsies are “not their thing” anymore. The reality is that the situation of the past will never return. Why is this so complex? One element is current medical curricula, from which pathology as a discipline has largely disappeared. Another is the spectacular improvement in modern medicine’s spectrum of diagnostic capabilities. Yet another is related to attitudes of attending physicians, of relatives of the deceased, and of pathologists who perform and report the autopsies. Recently many (usually more expensive) new autopsy approaches have been presented. It is unlikely that they will be able to reverse the tide of the decline, as long as there is no fundamental change in attitude towards autopsies, i.e., that attending physicians and pathologist no longer being convinced that autopsies are important as quality and teaching instrument.

In this paper, history, documented value, and reasons for the decline of the autopsy will be discussed, and suggestions will be made to turn the situation around.

## Short history of the autopsy

It is undeniable that the autopsy throughout history has been a key procedure in understanding the pathologic basis of disease and the cause of death of deceased patients. The first historical manuscript on this subject, not based on incidental observations but on a thorough study of disease progression, was written by Antonio Benivieni (1443–1502) and posthumously published by his brother Geronimo in 1507 as *De abditis nonnullis ac mirandis morborum et sanationum causis* (*About the hidden and incredible causes of diseases and cures*) [1]. This was an outstanding achievement at that time, confirmed by the fact that it lasted 170 years before the next book on this topic was published. That book (1679) by Theophile Bonet (1620–1689), *Sepulchretum sive anatomia practica ex cadaveribis morbo denatis* (*Burial vault*/*cemetery or anatomical studies on bodies affected by disease*) [2], was a compilation of nearly 3000 postmortem protocols written by more than 450 authors from the Hippocratic literature up to Bonet’s time. It provided further evidence in support of the notion that diseases might have an anatomical substrate.

The eighteenth century was the beginning of a golden period for the autopsy. The Dutch physician Herman Boerhaave (1668–1732) was the author of two monographs in which autopsies played a significant role. Both emphasized the importance of a good clinical history [3]. The autopsy reported in the first eventually led to recognition of the condition that is known as the “Boerhaave syndrome.” The other described a patient suffocating from a 3.5-kg mediastinal mass. These two observations, however, were overshadowed some 50 years later by Giovanni Morgagni’s *De sedibus et causis morborum per anatomen indignatis* (*About the seats and causes of diseases investigated by anatomical investigations*) in which he described 640 autopsies, systematically correlating the symptoms of his patients with the pathological findings at autopsy [4]. This was a breakthrough in the evolving concept that diseases have an anatomical substrate.

The man who definitely should be regarded as the father of the autopsy is the Austrian pathologist Carl von Rokitansky (1804–1878). He was the first who looked at pathological changes in human organs in a systematic manner. He carefully correlated morphology with clinical symptoms in developing the concept of pathogenesis. He was the first to take observations in Morgagni’s tradition to a higher level. This kind of work made him the best descriptive pathologist of his days [5].

In subsequent years, many pathologists paid increasing attention to details of autopsy techniques and to standardization of procedures, among them Virchow (1821–1902), who published a booklet on autopsy techniques that became widely used [6].

In the twentieth century, the autopsy flourished and laid a solid basis for understanding the pathogenesis of disease. But then from around 1970, the autopsy rate started to decline. The decline persisted and autopsy percentages dropped from about 50 % of hospital deaths around 1960 to approximately 10 %, and even less in some institutions, nowadays. Time will tell whether, after a period of 150 years, the medical autopsy has run its course as a useful method for investigating disease and establishing cause of death, other than in a medicolegal context.

## The value of the autopsy in the early 21st century

One might expect that the decrease in autopsy number has been at least partly due to decreasing enthusiasm among clinicians for this last examination. However, in the last 15 years, many papers were published that tell us otherwise. The number of papers, accumulated in PubMed in this period when using as search term “autopsy,” amounts to over 28,000 and for the two most recent years over 3800. Many of these papers concern case reports in which autopsy findings are reported. Research papers generally conclude that enough discrepancies between clinical and anatomical findings persist to warrant the performing of autopsies in the future. The discrepancies in the papers are usually classified according to the Goldman criteria [7]. A class I error is a major missed diagnosis, with potentially adverse impact on survival, that would have changed management. A class II error represents a missed major diagnosis, without potential impact on survival, that would not have changed therapy. The class III and IV errors are missed minor diagnoses not related to the cause of the main disease(s).

A powerful paper in this regard is the meta-analysis of Shojania et al. in JAMA in 2003 [8]. Their objective was to determine the rate at which autopsies detect important, clinically missed diagnoses and how this rate changed over time. To achieve this goal, the authors performed a systematic literature search for relevant English papers published between 1966 and 2002 and available in Medline. They identified 45 studies reporting 53 distinct autopsy series. Of the 53 series, 42 reported major errors, of which 37 class I errors according to the Goldman criteria. In 26 series, both major and class I errors were found. The authors estimated that, while the error rate had decreased, at the time of publication of their paper, US institutions would probably observe a major error rate of 8.4 to 24.4 % with a class I error rate of 4.1 to 6.7 %.

Wittschreiber et al. reported on 1800 adult autopsies at one single institution, the Charité Institute of Pathology in Berlin [9]. Randomly selected cases of the years 1988, 1993, 1998, 2003, and 2008 were analyzed. Based on power analysis, the rate of class I major discrepancies was 10.7 % in 2008, representing a reduction of 15.1 % relative to 1988.

A study from India [10] described the discrepancies between *antemortem* and *postmortem* diagnoses in 591 patients over the years 1947 to 2010 and concluded that the number of discrepancies had decreased significantly over time, but that the discrepancy rate in 2010 was still high at 9.3 %.

The same trend has been reported by several other groups studying unselected autopsy cases [11–13]. In studies of selected case series, the observed discrepancies tell a similar or an even worse story. A meta-analysis [14], which only included patients dying in an ICU in the period 1966 through 2011, found 31 studies reporting on a total of 5863 autopsies. In this study, in 28 % of the cases, at least one misdiagnosis was noted, 8 % of class I and 18 % of class II “despite presumably aggressive diagnostic assessment in the critical care environment.” They concluded “Our findings suggest that 34,000 ICU patients in the US may die as the result of a class I error annually, assuming that the error was the cause of death.” A Brazilian study [15] on critically ill patients with difficult antemortem diagnoses found in 50 % of 98 cases class I and class II discrepancies, of which cardiovascular complications were a main cause. An autopsy study in critically ill cancer patients in the ICU [16] found major discrepancies in 21 % of cases, with aspergillosis, pulmonary embolism, and cancer recurrence as the most commonly missed diagnoses. A study among pediatric patients requiring extracorporeal membrane oxygenator support found major discrepancies in 53 % of cases, the most commonly missed diagnosis being myocardial infarction found in 16 of 54 patients [17].

These and many other studies underscore the added value of the autopsy even in our era of relentless technical progress. We dare to postulate that the discrepancy rate between ante mortem and postmortem findings will remain at about 10 %.

## Autopsies as quality management tool

In the second half of the last century, the aspect “understanding the pathogenesis of disease and detecting new disease entities” has more or less faded into the background and several authors emphasized the role of the autopsy as quality assurance tool, focusing on the abovementioned differences between antemortem and postmortem diagnoses. In addition, autopsies continue to be considered valuable in medical education, e.g., providing case material for problem-based learning. Autopsy cases would provide students the opportunity to recall knowledge of anatomy and gain access to clinico-pathological correlations. The latter might contribute to a basis for quality management considerations.

The role of the autopsy in quality assurance concerns, among others, evaluation of the accuracy of diagnostic imaging, such as computerized tomography (CT), nuclear magnetic resonance (NMR), and positron emission tomography (PET) scans. Other fields of quality assurance are evaluation of efficacy and potential adverse effects of new drugs, new surgical techniques, and genetic engineering.

To this one might add activities in one way or the other related to quality, such as

- Detection of new patterns in old diseases (e.g., tuberculosis and syphilis)
- Providing information on disease course and cause of death to next of kin of a deceased patients
- Facilitating investigation of environmental, occupational, and lifestyle-related diseases
- Providing tissue for research
- Teaching medical students and residents in specialty training

The role of the autopsy as a quality management tool has focused on systematic analysis of discrepancies between clinical (antemortem) and autopsy (postmortem) diagnoses, e.g., Rosen et al. [18], Pastores et al. [19], and Tavora et al. [20]. Zampieri et al. recently re-emphasized the clinico-pathological conference, the quality of which often depends on whether or not an autopsy has been performed, as a worthwhile argument in maintaining the autopsy [21].

While autopsies conceptually contribute to auditing of aspects of medical care, to the best of our knowledge, no studies have shown that conducting autopsies and publicizing the findings are directly beneficial for patients [22]. No data have substantiated the notion that autopsy practice improves quality of patient care. This implies that any beneficial effect, in terms of quality assurance, of systematic performance of autopsies on quality of care remains a matter of faith and remains to be proven [23].

In spite of this lack of an evidence base, many clinicians and pathologists have experienced the teaching effect of an unexpected autopsy diagnosis, which may be beneficial for many patients to come, however difficult this might be to measure. Most commonly, autopsy reports reside in the obscurity of pathology and medical records departments as orphan data, because they are not systematically evaluated [22].

Moreover, one has to bear in mind that the autopsy itself is not error free since a pathologist, like any other physician, can and will occasionally render an erroneous diagnosis. No studies have been performed to determine error rates in autopsy diagnosis [24, 25]. In addition, a real danger exists for loss of quality in autopsy practice, along with decreasing pathologist experience in performing autopsies.

## Reasons for autopsy decline

While many blame the demise of the autopsy on a low rate of authorization by relatives or on a lack of interest of clinicians, this issue is quite a bit more complex. A variety of economic, social, medical, and technological factors are to be considered, and it would be too simple to look for a single cause or even a leading cause for this decline [26, 27]. Factors to be considered are as follows:

### Medical curricula

The last decennia, many medical schools have changed their curriculum from the classical discipline-based approach to an integrated system-based program. This has resulted in fragmentation of pathology teaching over many integrated courses, which tends to result in (much) fewer pathology contact hours than before. Time and facilities for autopsy and gross pathology demonstrations are often lacking. As a result, many students arrive at the end of the classroom phase of their medical studies without ever having attended an autopsy. With the low actual autopsy rate, many of them will never have attended an autopsy when they start their medical career. It is not difficult to imagine that for those, the autopsy is not a very relevant procedure and not the first issue to take care of when a patient has died.

### Clinicians’ attitude

With undergraduate curricula that do not provide a favorable climate for the autopsy, it cannot come as a surprise that many clinicians are skeptical about the need for requesting an autopsy. That might also be the reason why they are skeptical in regard of the reported levels of discrepancy between clinical and autopsy diagnoses and hence do not perceive the autopsy as an important procedure in clinical practice, despite the position of many professional organizations and learned societies and the wealth of evidence in support of the medical value of the autopsy [28]. New sophisticated diagnostic procedures have increased confidence in clinical diagnoses, resulting in the opinion that the autopsy is outdated and longer needed. Fear of litigation in case of a diagnostic error can exercise restraint as well [29]. In addition, advanced age of a deceased patient and the attitude of his or her relatives may negatively influence a clinician’s decision whether or not to request an autopsy. A reason frequently heard is the long turnaround time of final autopsy reports, which may take weeks but often months to be completed, to some extent depending upon the motivation of the responsible pathologist [30].

### Role of the pathologist

Many pathologists, in academic as in private practice, are quite vocal about their own dislike of autopsies. Some just do not like the procedure, which has been a reason for the Royal College of Pathologists (UK) to offer a training track for pathologists without developing any competency in autopsy practice. Others prefer alternative professional activities, in view of better remuneration or academic recognition (e.g., publishing). New molecular findings in research and predictive diagnostics offer more opportunities for publishing high-impact papers and are more instrumental in developing a career in academic pathology.

When asked, many pathologists will still provide lip service to the importance of autopsies but fail to develop initiatives in their own hospital to increase the autopsy rate. On the priority list, autopsies and autopsy reports end up at the bottom. It will not come as a surprise that in many residency training programs, the novice resident starts training with a rotation in the autopsy room. And yet, the autopsy is the most complex technical procedure of our discipline, requiring high-level theoretical and practical anatomy and pathology knowledge as well as a good level of understanding of histopathology. Why then would this field of activity be preferentially assigned to the least experienced members of staff? While medical education has undergone important changes in the last half century, training programs in pathology often still blindly follow a tradition that has its roots back in the nineteenth century.

The confrontation with junior residents in the autopsy room as a rule does not motivate the involved clinicians. In this sense, it is revealing that autopsy pathology is not yet an officially recognized subspecialty in pathology; worse even, subspecialization is not even discussed presently, in spite of the serious problems that are encountered. The declining autopsy rate is evidently not an issue of primary concern.

### Financial reasons

Financial reasons are often stated as a reason for autopsy decline and there is a lot of truth in this suggestion. A complex autopsy case will include consulting a variety of specialists and substantial reporting which might include a clinico-pathological conference which will require many hours (maybe even a full day) if the pathologist takes the case seriously. Financial compensation usually falls short of what would be gained by reporting surgical specimens in the same time frame, which is in addition perceived as more convenient. One reason for this unsatisfactory compensation is that for hospital management, the honorarium for the pathologist is but part of the total cost of an autopsy, which as a rule exceeds a few thousand dollars/euros. Autopsy cost is often not reimbursed by health insurance companies as they tend to claim that a deceased patient is not their client anymore. Adequate funding for autopsies is likely to improve the interest for the autopsy. [31].

### Role of the family

The reasons most commonly cited by clinicians for the decline in the autopsy rate include an increasingly onerous consent process and the assumption that bereaved family members are hostile to the idea of an autopsy. In a recent survey in Germany, about 50 % of people participating in the survey considered autopsy a necessary procedure and would allow their own relatives to be autopsied. In a German study, many who had recently experienced the death of a relative in a hospital had not been asked permission for an autopsy [32]. In communicating with clinicians on the issue of authorization for autopsy, the perceived percentage of refusal ranged from 50 to 90 %. Close scrutiny suggested that many clinicians did not request permission but up-front declared that the relatives denied autopsy. The increasingly different ethnic backgrounds and religious convictions in our increasingly multicultural society contribute to the factual reasons for refusal of permission for autopsy. An open question is whether or not the autopsy permission rate would increase if relatives would be offered an alternative non-invasive autopsy procedure. Moreover, patients often have the impression that they do the doctor a favor by granting permission for an autopsy, rather than realizing that it is their right to have this final consultation to confirm that diagnoses and treatment were conducted *lege artis*.

What follows from the situation described under “financial reasons” is that relatives who would tend to insist on an autopsy on a deceased family member, for whichever reason, might be confronted with a significant invoice from the hospital, which can make them refrain from asking for an autopsy or from pursuing their request. Our discipline should come up with innovative approaches for funding an autopsy in situations in which payment by relatives is not reasonable.

## New approaches to autopsy procedure

In view of the major objections of relatives against a classical autopsy, alternative autopsy techniques have been conceived which might overcome this problem. In the last decade, other approaches to achieve this goal have been developed. A variety of imaging approaches, often combined with targeted biopsies, have been published. To list a few, we can mention the minimally invasive autopsy, the needle autopsy, the endoscopic autopsy, the imaging autopsy, the verbal autopsy, the virtual autopsy, and the partial autopsy [33–36]. An advantage of minimally invasive procedures appears to be a better quality of the obtained tissue samples relative to those taken during the conventional autopsy, which constitutes an additional reason to pursue these techniques [37].

These alternative autopsy techniques employing “clean” and familiar imaging techniques (which can obtain adequate samples for histological analysis) deserve a place in pathology and might increase the number of autopsies performed in hospitals. However, the success of this approach will again depend strongly on the clinician’s conviction of the value of the autopsy. Clinicians need to convince the relatives and this will be less successful if the clinician himself lacks in conviction.

It is difficult to predict the cost of alternative autopsy procedures as this depends on a variety of factors. But even if the nominal cost might be less, their introduction might increase the overall cost of autopsy services. According to Thayyil, “Although minimally invasive autopsy can be less expensive than conventional autopsy, the overall cost of postmortem services might rise because parents (relatives) who decide against autopsy might want a minimally invasive autopsy, which paradoxically increases the workload for both pathologists and radiologists” [33].

## Indications for performing an autopsy

Autopsies are important in quality management, teaching, training, tissue collection for research (when permitted), death statistics, etc. However, in this era, when autopsies seem to almost vanish, an autopsy can be mandatory under certain circumstances. A list of these indications, as published by the German Pathology Society, might be of help [38]. This list mostly stipulates medicolegal situations which are beyond the subject of this paper. Many clinical situations strongly calling for an autopsy are not listed.

## The future of the autopsy in our hands

Autopsies have been on the decline in the past 50 years. It is not realistic to think that we can turn this tide. Key concepts regarding the pathogenesis of disease have come from autopsy observations in the past. Practicing physicians rely on this knowledge and may continue to learn from autopsies conducted on their own patients, which generates experience used in daily practice. In addition, in spite of the wealth of new diagnostic laboratory and imaging techniques which often seem to make an autopsy superfluous, errors are still made. Autopsy as the “final consultation” before a patient file is closed remains a valuable tool for self-evaluation of performance, and unexpected autopsy findings continue to provide life-long learning moments. However, error rates have not gone up since the autopsy rate started to decline and solid evidence that quality of care has suffered does not exist.

There is no doubt that pathologist attitude towards autopsies has changed dramatically: lack of interest and perception of an autopsy as a burden without significance or even as inferior work. The position of the autopsy tends to be played down when pathologists discuss what they do with outsiders. Lack of interest results in delayed reports that often do not or only partially provide relevant answers to the clinicians’ questions and some conclusions even end up to be wrong. Many practicing pathologists lack sufficient up-to-date pathophysiological knowledge and clinical orientation to adequately understand and answer the questions asked by clinicians. The latter is a reflection of insufficient emphasis on understanding mechanisms of disease (general pathology) in many training programs, with their strong focus on diagnostic subspecialty competences.

The decline of the autopsy rate is a reality, and with the limited number performed, it is increasingly difficult to acquire sufficient experience in performing, interpreting, and reporting autopsies. It is essential that pathologists who perform autopsies are enthusiastic, interested, and competent and respected for their knowledge in this field of our discipline. Only these qualities will make them appreciated partners of clinicians and good teachers of our residents. The only way to achieve this goal is subspecialization in clinical autopsy pathology, much like what has developed for forensic pathology [39, 40]. Training in this subspecialty should include clinical training in intensive care medicine, because ICU cases provide the most challenging questions. Knowledge of this field will allow them to become real “sparring partners” for intensivists, much like fetoplacental and neuropathologists who are more readily accepted by their clinical colleagues as indispensable partners. Training autopsy pathologists for all subspecialties would be an illusion, and, the ICU being the *ultimum refugium* for patients of most disciplines, the clinical autopsy pathologist will remain confronted with a wide range of diseases.

The creation of autopsy pathology as a subspecialty might also solve the issue of late and insufficient reporting. The subspecialist will develop and introduce new autopsy techniques and will develop initiatives to change the attitude towards autopsies in the clinics. If we bring our best human capital to the frontline and respect these professionals as highly as any other subspecialist, the tide may change, but only if we really want to.
